# Colon cancer metastasis mimicking a hilar cholangiocarcinoma: a case report and literature review

**DOI:** 10.1186/s40792-020-00992-w

**Published:** 2020-09-25

**Authors:** Takashi Ofuchi, Hiromitsu Hayashi, Takanobu Yamao, Takaaki Higashi, Toru Takematsu, Yosuke Nakao, Kensuke Yamamura, Katsunori Imai, Yo-ichi Yamashita, Hideo Baba

**Affiliations:** Department of Gastroenterological Surgery, Graduate School of Life Sciences, Kumamoto University, 1-1-1 Honjo, Chuo-ku, Kumamoto, 860-8556 Japan

**Keywords:** Colon cancer metastasis, Hilar cholangiocarcinoma immunohistochemical staining

## Abstract

**Background:**

An accurate diagnosis of the primary cancer or metastatic tumor is quite important because misdiagnosis may lead to the selection of incorrect adjuvant therapy and worse long-term outcomes after surgery. Here, we present a rare case with an unusual metastatic pattern mimicking a hilar cholangiocarcinoma originated from colon cancer

**Case presentation:**

A 69-year-old man was referred to our hospital because of an upper bile duct stenosis. He had the past history of the sigmoidectomy for the primary colon cancer and the partial hepatectomy with radiofrequency ablation (RFA) for synchronous liver metastases 4 years ago. After 2 years from the initial operation, he had experienced the local recurrence of post-RFA lesion and had undergone a partial hepatectomy. According to the radiological findings of the bile duct stenosis, we diagnosed a hilar cholangiocarcinoma (Bismuth type II), and then he underwent the extended right hepatectomy with extrahepatic bile duct resection. Histological findings including the immune-histochemical examinations (CK7−, CK20+, CDX2+ and SATB2+) uncovered the metastatic tumor into extrahepatic bile duct originated from the primary colon cancer.

**Conclusion:**

We experienced an extremely rare case with a colon cancer metastasis mimicking a hilar cholangiocarcinoma. In this case with a past history of colon cancer, an immunohistochemical staining led us to distinguish the primary hilar cholangiocarcinoma and the mimicking tumor.

## Introduction

Metastasis of colon adenocarcinoma is commonly found in the lung, liver, or peritoneum. Metastasis to the extrahepatic bile duct is an extremely rare manifestation of colon cancer. An accurate diagnosis of the primary or metastatic cancers is quite important because misdiagnosis may lead to the selection of incorrect adjuvant therapy and worse long-term outcomes after surgery. Here, we present a case of colon cancer with an unusual metastatic pattern mimicking a hilar cholangiocarcinoma.

## Case presentation

**Fig. 1:**
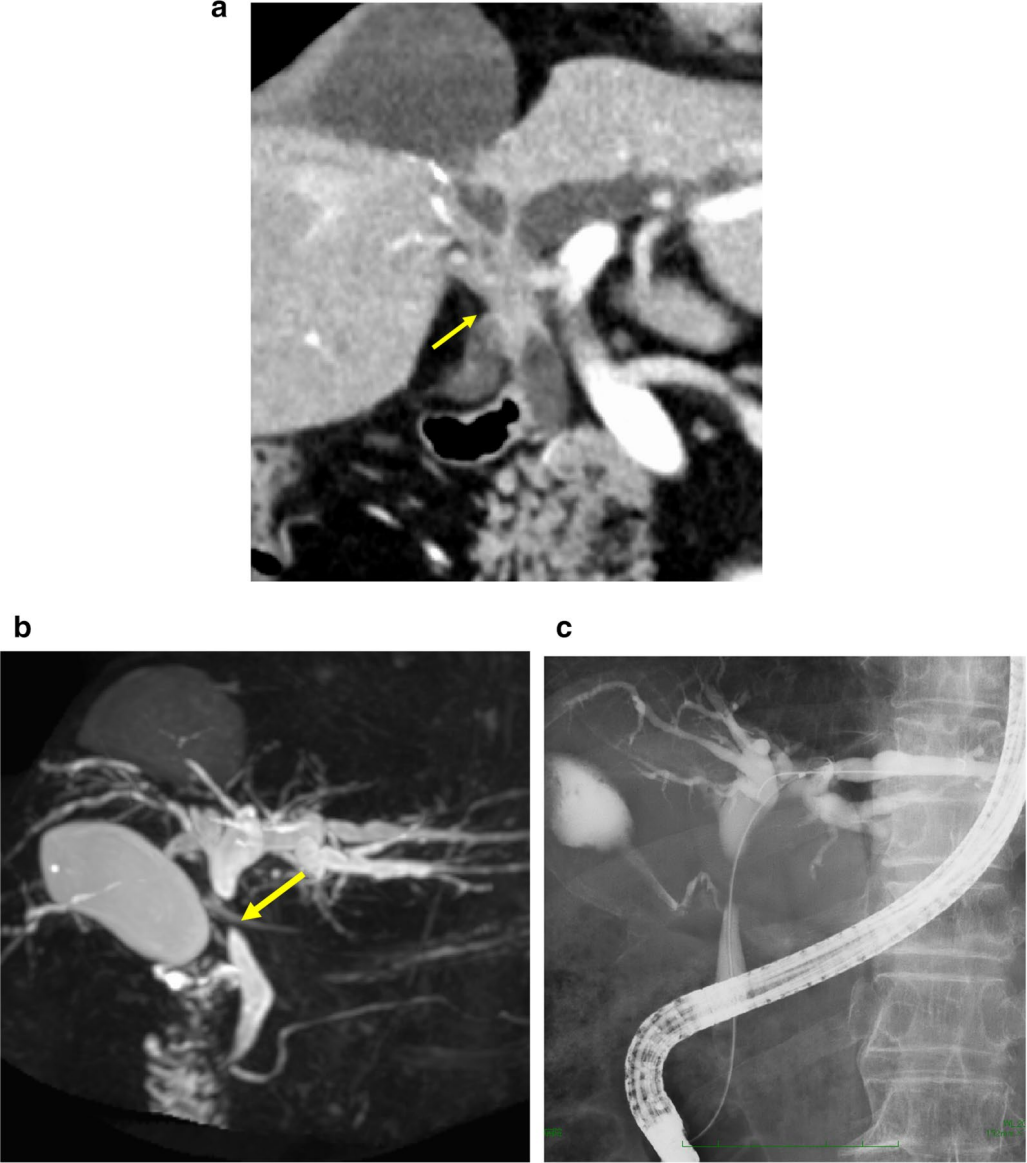
**a** Enhanced CT revealed an enhanced wall thickness in the hilar bile duct. **b**, **c** Magnetic resonance cholangiography, endoscopic retrograde cholangiography and intraductal ultrasound showed that the tumor had grown into the bile duct lumen

**Fig. 2:**
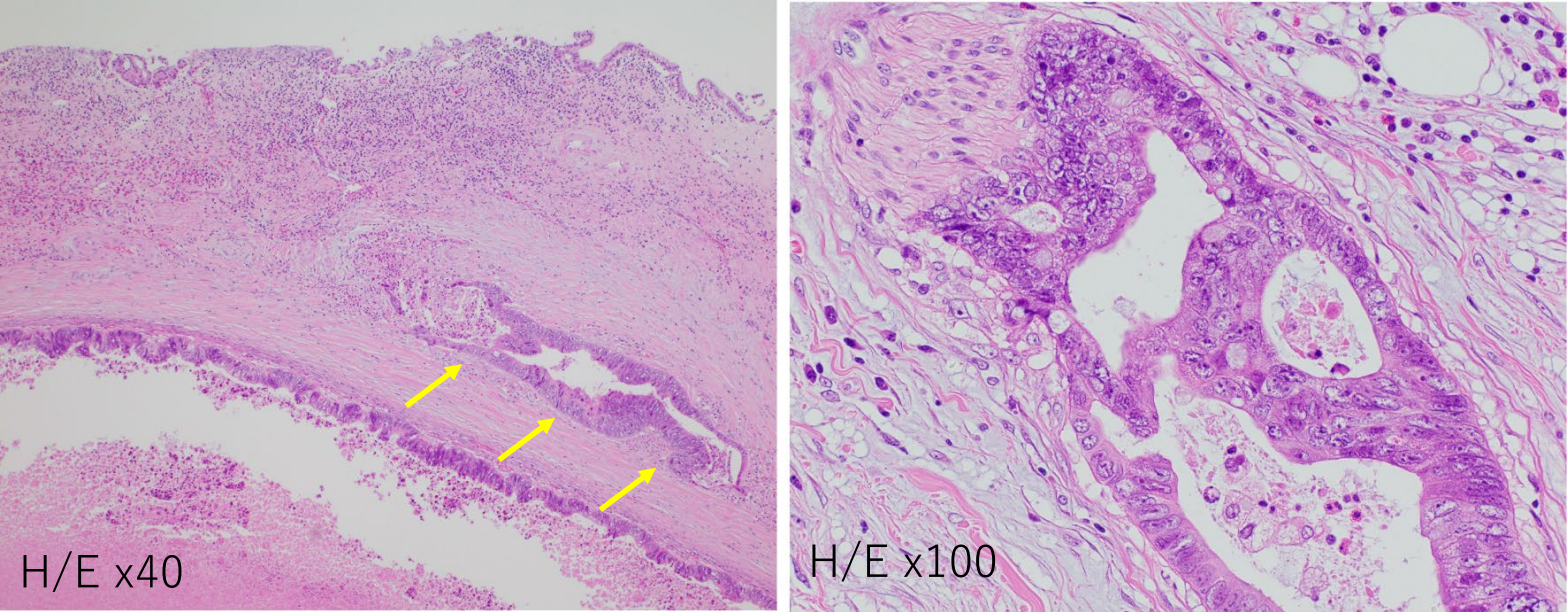
Microscopic findings of colon cancer metastasis mimicking hilar cholangiocarcinoma. Their arrows indicate perineural infiltration

**Fig. 3:**
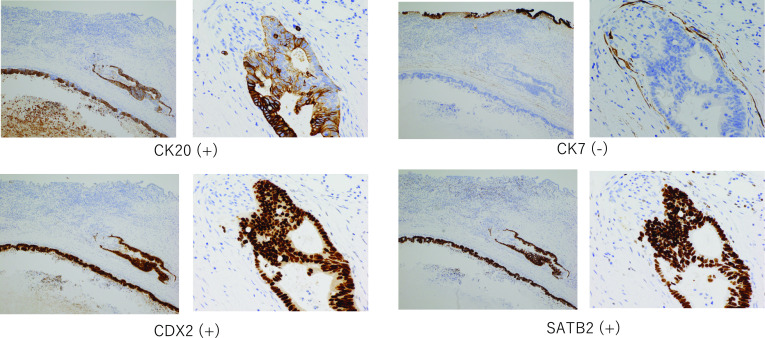
Immunohistological findings of colon cancer metastasis mimicking hilar cholangiocarcinoma

A 69-year-old man had the past history of laparoscopic sigmoidectomy for pathological stage IV colon cancer with synchronous liver metastases (Segment 7 and 8) 4 years ago. He underwent sigmoidectomy due to intestinal obstruction, and liver lesions were planned for a two-stage surgery. Histological examination of the primary tumor revealed a well-differentiated adenocarcinoma with subserosal invasion and vascular infiltration, but no lymphatic or lymph node metastasis. Thereafter, he had undergone partial hepatectomy of Segment 7 lesion and radiofrequency ablation (RFA) of Segment 8 lesion for colorectal liver metastases 2 months after sigmoidectomy. After 2 years from the initial operation, he had undergone partial hepatectomy for the local recurrence of post-RFA lesion. He was followed without any adjuvant chemotherapy since the resection of primary colon cancer. After that, he was referred to our hospital because of the stenosis in a hilar bile duct. Enhanced CT revealed an enhanced wall thickness in the hilar bile duct (Fig. 1a). Magnetic resonance cholangiography, endoscopic retrograde cholangiography and intraductal ultrasound showed that the tumor had grown into the bile duct lumen (Fig. 1b, c). A brush cytology appearance indicated a class IV. According to the radiological findings, we diagnosed a hilar cholangiocarcinoma (Bismuth type II). Then, he underwent a right portal vein embolization and followed by the extended right hemihepatectomy with extrahepatic bile duct resection. The macroscopic finding displayed 3 cm tumor located in the hilar bile duct wall. Histological examination revealed moderately differentiated adenocarcinoma existing in the common hepatic duct. There is no tumor invasion into the right hepatic artery. Perineural infiltration was observed, but portal or vascular infiltration or lymph node metastases were not identified. There is no invasion of intraepithelial neoplasia at the margin of the tumor (Fig. 2). The immunohistological stainings of the bile duct tumor showed the cytokeratin (CK)7-negative, CK20-positive, caudal-type homeobox 2 (CDX2)-positive and special AT-rich sequence-binding protein (SATB2)-positive, uncovering that the bile duct tumor originated from the sigmoid colon carcinoma (Fig. 3).

## Discussion

Primary cholangiocarcinoma is a typical malignant disease that causes obstruction of the extrahepatic bile duct. Bile duct obstruction due to metastasis of extrahepatic bile duct in gastric cancer and metastasis to lymph nodes around the bile duct in lung cancer has been reported [1], but extrahepatic bile duct metastasis of colorectal cancer is extremely rare. An accurate diagnosis of the primary cholangiocarcinoma or metastatic tumor is quite important because misdiagnosis may lead to the selection of incorrect adjuvant therapy and worse long-term outcomes after surgery. Differential diagnosis between primary cholangiocarcinoma and bile duct metastasis of colorectal cancer is difficult with preoperative imaging and hematoxylin–eosin staining. In this case, it was difficult to distinguish between primary cholangiocarcinoma and colon cancer metastasis using preoperative radiological findings and HE staining.

Recently, the usefulness in confirming the diagnosis of immunostaining with CK7, CK20, and CDX20 has been reported. Rullier et al. reported that the ratio of CK7(−)/CK20(+) was 4% in bile duct cancer, but 81% in colorectal cancer, and a combination of CK7 and CK20 is useful to determine that the ductal tumor was a metastatic lesion arising from colon cancer [2]. Furthermore, CDX2 and SATB2 are a highly sensitive and specific markers for metastatic lesions of gastrointestinal tract adenocarcinoma, especially colon cancer [3].

In this case, the results of immunohistological staining of the tumor were a cytokeratin CK7-negative, CK20-positive, CDX2-positive, SATB2-positive histologically intraepithelial neoplasia was not found at the margin of the tumor. From these findings, we diagnosed a bile duct metastasis of the sigmoid colon carcinoma.

In PubMed (keyword of “colon cancer”, “bile duct metastasis”), there were only 11 cases from 2007 to 2018. 7 of these cases have detailed descriptions in these reports [4–9]. Table 1 summarizes 8 cases including our case. The histological type was moderately differentiated in 7 cases, 1 case had the pancreaticoduodenectomy, 2 cases had the extended right hepatectomy, and 1 case had the pancreaticoduodenectomy and extended right hepatectomy. In all cases, an immunohistological staining is useful to diagnose the bile duct metastasis from colon cancer. In our case, we used SATB2 to diagnose a bile duct metastasis of the sigmoid colon carcinoma. In addition, there is a possibility of recurrence due to incomplete RFA in the present case.

**Table 1:** Summary of reported cases of bile duct metastasis from colon cancer

Case	Author	Age/sex	Recurrence period (month)	Tumor site	Therapy	Immunohistochemical staining	Liver metastasis
1	Yamao	65/F	94	Distal bile duct	PD	CK7(−) CK20(+)	None
2	Lee	70/M	0	Distal bile duct	PD+ extended right hemihepatectomy	CK7(−) CK19(+) CK20(+) CDX2(+)	None
3	Kawakatsu	61/M	108	Hilar bile duct–right hepatic duct	Extended right hemihepatectomy	CK7(−) CK20(+)	None
4	Strauss	57/M	–	Distal–hilar bile duct	Endoscopic biliary stenting	CK20(+) CDX2(+)	None
5	Koh	–	–	Distal bile duct	Endoscopic procedure	CK7(−) CK20(+)	–
6	Koh	–	–	Confluence of hepatic ducts	Endoscopic procedure	CK7(−) CK20( +)	–
7	Knowles	50/M	19	Confluence of hepatic ducts	Endoscopic biliary stenting	CK7(−) CK20(+) CDX2(+)	None
8	This case	69/M	48	Hilar bile duct	Extended right hemihepatectomy	CK7(−) CK20(+) CDX2(+) SATB2(+)	Segment 7 and 8 (resection and RFA)

There are several possible metastatic routes to the bile duct from the primary colon cancer or liver metastasis: (1) arterial hematogenous, (2) direct infiltration through peribiliary capillary plexus, (3) lymphatic, (4) implantation of cancer cells [10, 11], and (5) dissemination associated with RFA.

In this case, there are two possible metastatic routes: (1) arterial hematogenous, (5) dissemination associated with RFA. Although a part of the tumor was exposed on the bile duct mucosal surface, most of bile duct epithelium was the non-neoplastic component. Also, the tumor invaded into the adipose tissue around the bile duct wall with perineural infiltration. These findings suggest the possibility of arterial hematogenous as a metastatic route. On the other hand, this patient had experienced RFA for liver metastasis and the subsequent recurrence. We cannot exclude the possibility of intravascular or intraductal dissemination due to incomplete RFA as a metastatic route.

## Conclusions

This is a rare case of colon cancer with an unusual metastatic pattern mimicking a hilar cholangiocarcinoma. Immunostaining with CK7, CK20, CDX20, SATB2 is useful to distinguish the ductal tumor was a primary lesion or a metastatic lesion arising from colon cancer. In the case with a bile duct tumor and a past history of primary colon cancer or liver metastasis, the colon cancer metastasis mimicking a bile duct tumor could be possible.

## Data Availability

All data generated or analyzed during this study are included in this published article.

## Abbreviations

RFA: Radiofrequency ablation
CT: Computed tomography
CK: Cytokeratin
CDX2: Caudal-type homeobox 2
SATB2: Special AT-rich sequence-binding protein 2

## Presentation

We have not submitted this manuscript anywhere.
